# Modelling soil detachment by overland flow for the soil in the Tibet Plateau of China

**DOI:** 10.1038/s41598-019-44586-5

**Published:** 2019-05-30

**Authors:** Mingyi Li, Xiao Hai, Huan Hong, Yanyan Shao, Doudou Peng, Wennian Xu, Yueshu Yang, Yan Zheng, Zhenyao Xia

**Affiliations:** 1 Key Laboratory of Geological Hazards on Three Gorges Reservoir Area (China Three Gorges University), Ministry of Education, Yichang, 443002 People’s Republic of China; 2 Engineering Research Center of Eco-environment in Three Gorges Reservoir Region, Ministry of Education, China Three Gorges University, Yichang, 443002 People’s Republic of China; 3 Collaborative Innovation Center for Geo-hazards and Eco-environment in Three Gorges Area of Hubei Province, China Three Gorges University, Yichang, 443002 People’s Republic of China; 4 College of Biological and Pharmaceutical Sciences, China Three Gorges Univiersity, Yichang, 443002 People’s Republic of China; 5 School of Architectural Engineering, Tibet Vocational Technical College, Lhasa, 850030 People’s Republic of China

**Keywords:** Hydrology, Solid Earth sciences

## Abstract

The overland flow erosion is common and became more serious because of the climate warming inducing more runoff in the Tibet Plateau. The purposes of this study were to evaluate the effects of flow rate, slope gradient, shear stress, stream power, unit stream power and unit energy of water-carrying section on the soil detachment capacity for the soil in the Tibet Plateau of China due to the information is limited. To achieve this aim, laboratory experiments were performed under six flow rates (5, 10, 15, 20, 25 and 30 L min^−1^) and six slope gradients (8.74%, 17.63%, 26.79%, 36.40%, 46.63 and 57.73%) by using a slope-adjustable steel hydraulic flume (4 m length, 0.4 m width, 0.2 m depth). The results indicated that soil detachment capacity ranged from 0.173 to 6.325 kg m^−2^ s^−1^ with 1.972 kg m^−2^ s^−1^ on average. The soil detachment capacity increased with power function as the flow rate and the slope gradient augmented (R^2^ = 0.965, NRMSE = 0.177 and NSE = 0.954). The soil detachment capacity was more influenced by flow rate than by slope gradient in this study. The relation between soil detachment capacity and shear stress, stream power, unit stream power and unit energy of water-carrying section can be described by using the linear function and power function, the power function relationship performed better than the linear function in generally. The stream power exhibits the best performance in describing the soil detachment capacity among shear stress, stream power, unit stream power and unit energy of water-carrying section in this study. The erodibility value in this study was larger than and the critical shear stress was less than those for soil in the eastern China. There has a huge potential for the soil in the Tibet Plateau eroded by the water erosion when enough runoff exiting. More attention should be payed to the water erosion process and mechanism in the Tibet Plateau area in the future.

## Introduction

The increase in temperature caused by climate warming has been paid more and more attentions in recent several decades^1–3^. The global average surface temperature has increased by 0.65 to 1.06 °C (0.85 °C in average) over 1880–2012^4^. In China, the average surface temperature has increased by 1.40 °C during 1951–2007, with a changing rate of 0.25 °C (10 yr)^−1^, indicating an accelerating warming trend in recent decades^5^. The Tibetan Plateau, the largest and highest geoform on the Eurasian continent that covers an area of about 2.5 million km^2^ with a mean elevation of about 4000 m a.s.l^6^, has been subjected to significant climate warming over the last few decades and suffered a relatively faster warming rate ranging from 0.20 °C (10 yr)^−1^ to 0.50 °C (10 yr)^−1 5^. This intensifies the thawing for ice and snow, leading to a low/lack snow pack and more rainfall, and resulting in more overland flow, can significantly aggravated soil erosion^2,7^. Serious erosion would be induced by a small amount of overland flow after thawing^8^.

Soil detachment refers to the dislodging of soil particles and aggregates from the soil matrix by erosive agents at particular locations on the soil surface, thereby creating loose, non-cohesive sediments for subsequent transport and deposition^9–11^. Soil detachment by overland flow (e.g. rill, ephemeral gully and gully erosion) is the dominant process of soil erosion^12–14^. Soil detachment capacity can be defined as the maximum soil detachment rate under clear overland flow condition based on the sediment feedback relationship between soil detachment rate and sediment load in overland flow^15,16^. Flow hydraulic conditions could affected the soil detachment capacity as they were the driving force for soil detachment^14,17^. Both slope gradient and flow rate could alter flow energy thus influence the soil detachment capacity for the given kind of soil^18^. The soil detachment capacity, a basic and key parameter in process-based erosion models, could be expressed by flow hydrodynamic parameters (e.g. shear stress, stream power, unit stream power and unit energy of water-carrying section) in different soil erosion model and by different researchers^19–23^. However, the effects of flow rate, slope gradient, shear stress, stream power, unit stream power and unit energy of water-carrying section on the soil detachment capacity for the soil in the Tibet Plateau of China remains unknown.

Soil properties has profound effects on the soil detachment capacity as they could affect the soil detachment process^24^. Soil detachment capacity is expressed as a linear function with parameters of soil erodibility and the critical shear stress^15^. Both soil erodibility and the critical shear stress are mainly influenced by soil properties. Hanson and Robinson^25^ suggested that soil erodibility was considerable affected by soil bulk density. Sheridan *et al*.^26^ demonstrated the rill erodibility was positively related to the proportion of particles in the 0.02–1.0 mm range while negatively correlated with particle classes <0.02 mm and higher than about 5–10 mm. Geng *et al*.^27^ highlighted the significant importance of clay content, sand content and the median soil grain size on rill erodibility. Wang *et al*.^17^ confirmed the rill erodibility closely related to soil cohesion, clay content and the median soil grain size. Liu *et al*.^28^ underlined the soil erodibility was impacted by the clay content and the soil void ratio. Wang *et al*.^29^ and Xiao *et al*.^30^ determined the effect of aggregate stability on the rill erodibility. The soil organic matter can also influence the soil erodibility due to its promotion in the development of soil aggregates^31^, The critical shear stress also demonstrated to have close relationships with the soil physicochemical properties^17,28,29^. Numerous studies have investigated the effects of soil properties on the soil detachment capacity, soil erodibility and the critical shear stress. However, the information on these parameters is seriously limited for the soil from the Tibetan Plateau^32^, where the dominant soil textures are characteristic of a low degree of development, parent materials, coarse particles, and detritus particle distribution^33^.

Against this background information, this study was conducted to model soil detachment by overland flow for the soil in the Tibet Plateau of China. The purposes of this study were (i) to analysis influence of slope gradient and flow rate on soil detachment capacity; (ii) to estimate the relationship between soil detachment capacity with flow hydrodynamic parameters and (iii) to obtain the soil erodibility and the critical shear stress for the soil in the Tibet Plateau of China.

## Results

### Influence of slope gradient and flow rate on soil detachment capacity

**Figure 1:**
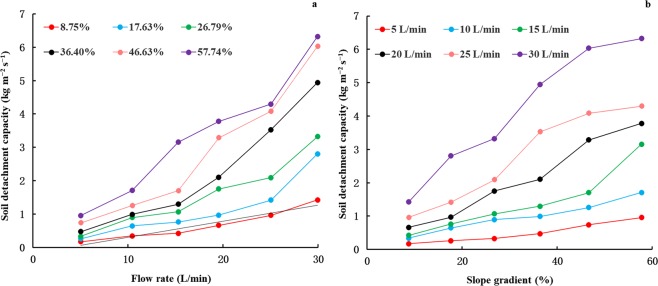
Relationships between soil detachment capacity and (**a**) flow rate; (**b**) slope gradient.

Soil detachment capacity greatly increased as the overland flow rate and slope gradient augmented (Fig. 1). The soil detachment capacity ranged from 0.173 to 6.325 kg m^−2^ s^−1^ with 1.972 kg m^−2^ s^−1^ on average, which demonstrated significant positive correlations with the overland flow rate and slope gradient (P < 0.01) (Table 1). Power function could well describe the relationship of soil detachment capacity with both overland flow rate and slope gradient (Table 2). The R^2^ value ranged from 0.929 to 0.984, all of them were higher than 0.90, indicating that these fitting equations can explain more than 90% of the variance in the soil detachment capacity. The NRMSE ranged from 0.073 to 0.316, most of which were less than 0.2, illustrating these fitting equations can describe the soil detachment capacity with small relative residuals. The NSE ranged from 0.806 to 0.971, all of them were larger than 0.70, suggesting a good performance of these fitting equations. The values of value of *R*^2^, *NRMSE* and *NSE* for slope gradient were larger than those for runoff rate in most case, demonstrating the slope gradient can be more effectively describe the soil detachment capacity than runoff rate.

**Table 1:** Pearson correlation coefficients for the soil detachment capacity related to slope gradient, flow rate, shear stress, stream power, unit stream power, and unit energy of water-carrying section.

	Q	S	τ	ω	P	E
Dr	0.726**	0.577**	0.938**	0.973**	0.818**	0.944**

Dr is soil detachment capacity (kg m^−2^ s^−1^); Q is flow rate (L min^−1^); and S is slope gradient (%); τ is shear stress (Pa); ω is stream power (N m^−1^ s^−1^); P is unit stream power (m s^−1^); E is unit energy of water-carrying section (m), ** Significant at 0.01 level of probability.

**Table 2:** Correlation Coefficients between soil detachment capacity and slope gradient and flow rate and the statistical evaluation of the performance of their relationships.

Slope gradient (%)	Fitting equation	R^2^	NRMSE	NSE	Flow rate\(L min^−1^)	Fitting equation	R^2^	NRMSE	NSE
8.75	Dr = 0.024 Q ^1.138^	0.957	0.187	0.914	5	Dr = 0.021 S ^0.901^	0.941	0.159	0.921
17.63	Dr = 0.036 Q ^1.180^	0.929	0.316	0.806	10	Dr = 0.062 S ^0.799^	0.984	0.078	0.969
26.79	Dr = 0.046 Q ^1.220^	0.977	0.138	0.949	15	Dr = 0.049 S ^0.960^	0.943	0.244	0.850
36.40	Dr = 0.050 Q ^1.295^	0.958	0.190	0.927	20	Dr = 0.072 S^0.965^	0.970	0.094	0.969
46.63	Dr = 0.092 Q ^1.177^	0.943	0.173	0.928	25	Dr = 0.133S^0.871^	0.964	0.105	0.951
57.74	Dr = 0.169 Q ^1.040^	0.980	0.100	0.963	30	Dr = 0.255 S^0.809^	0.983	0.073	0.971

Dr is soil detachment capacity (kg m^−2^ s^−1^); Q is flow rate (L min^−1^); and S is slope gradient (%); R^2^ is determination coefficient; NRMSE is normalized root mean square error and NSE is Nash-Sutcliffe efficiency index.

The combined influence of slope gradient and flow rate on the soil detachment capacity was performed by the multivariate non-linear regression analysis, suggesting the power function can effectively describe their relationship (Equation 1).

1 $$Dr=0.002{S}^{0.867}{Q}^{1.361}\,\,\,\,\,\,\,{{\rm{R}}}^{{\rm{2}}}=0.965\,\,\,\,\,{\rm{n}}=36$$

where *S* is the slope gradient (%) and *Q* is the flow rate (L min^−1^). The exponent for flow rate is 1.361, 55.37% larger than that for slope gradient, indicating that the soil detachment capacity is more influenced by flow rate than by slope gradient in this study. The *R*^2^, *NRMSE* and *NSE* derived from Equation 1 were 0.965, 0.177 and 0.954, respectively, indicating that Equation 1 can explain 96.5% of the variance in the soil detachment capacity with small relative residuals and has a good performance (>0.7).

### Relationship between soil detachment capacity and flow hydrodynamic parameters

**Figure 2:**
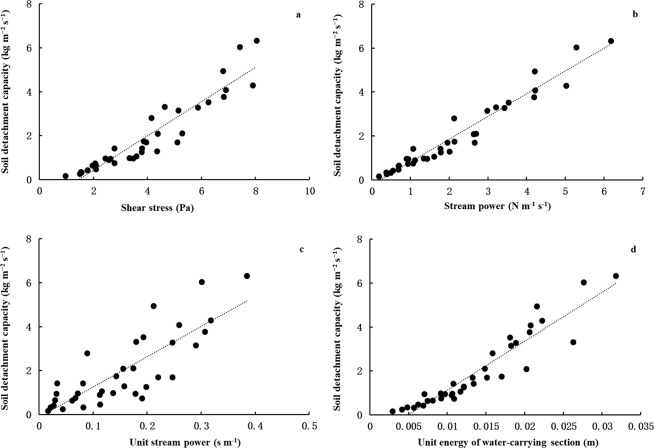
Relationship of soil detachment capacity with the flow hydrodynamic parameters.

The soil detachment capacity increased pronouncedly with the increase in flow hydrodynamic parameters (Fig. 2). Pearson correlation coefficients demonstrated the soil detachment capacity was significant positive correlations with all the flow hydrodynamic parameters in this study (P < 0.01) (Table 1). The linear function and power function relationship were tried to understand the relationship between soil detachment capacity and flow hydrodynamic parameters due to they were effective in most case^11,21,29^. The *R*^2^ ranged from 0.669 to 0.946 and from 0.627 to 0.956, the *NRMSE* ranged from 0.192 to 0.467 and from 0.203 to 0.541, and the *NSE* ranged from 0.690 to 0.946 and from 0.572 to 0.940 for the linear function and power function relationship, respectively (Table 3). The shear stress, stream power and unit energy of water-carrying section could effectively describe the soil detachment rate with good performance, while that for the unit stream power only performed with satisfactory performance for both linear function and power function relationship. Generally, the power function relationship performed better than the linear function for describe the relationships with an exception for unit stream power. The stream power performed the best in describing the soil detachment capacity, followed by the shear stress / the unit energy of water-carrying section and the unit stream power was the worst performance according to the values of the statistical parameters of *R*^2^, *NRMSE* and *NSE* (Table 3).

**Table 3:** Statistical evaluation of the performance of the relationship between soil detachment capacity and hydrodynamic parameters.

Flow hydrodynamic parameters	Linear function relationship				Power function relationship			
	Fitting equation	R^2^	NRMSE	NSE	Fitting equation	R^2^	NRMSE	NSE
*τ*	Dr = 0.780 *τ*−1.138	0.880	0.287	0.880	Dr = 0.173 *τ* ^1.651^	0.938	0.258	0.903
*ω*	Dr = 1.035 *ω*−0.225	0.946	0.192	0.946	Dr = 0.848 *ω* ^1.058^	0.956	0203	0.940
*P*	Dr = 13.764 *P*−0.125	0.669	0.467	0.670	Dr = 8.705 P ^0.858^	0.627	0.541	0.572
*E*	Dr = 222.791 *E* – 1.095	0.885	0.272	0.892	Dr = 1652.880 P ^1.605^	0.941	0.260	0.901

Dr is soil detachment capacity (kg m^−2^ s^−1^); τ is shear stress (Pa); ω is stream power (N m^−1^ s^−1^); P is unit stream power (m s^−1^); E is unit energy of water-carrying section (m); R^2^ is determination coefficient; NRMSE is normalized root mean square error and NSE is Nash-Sutcliffe efficiency index.

### The rill erodibility and critical flow hydrodynamic parameters

The rill erodibility and critical flow hydrodynamic parameters can be estimated from the slope of the regression line and the intercept on the x-axis for the linear function^15^. Therefore, in this study, the erodibility values based on shear stress, stream power, unit stream power and unit energy of water-carrying section were 0.780 s m^−1^, 1.035 s^2^ m^−2^, 13.764 kg m^−3^ and 222.791 kg m^−3^ s^−1^, respectively. And the corresponding critical shear stress, critical stream power, critical unit stream power and critical unit energy of water-carrying section were 1.459 Pa, 0.217 N m^−1^ s^−1^, 0.908 cm s^−1^ and 0.491 cm, respectively.

## Discussion

The soil detachment capacity was more sensitive to flow rate than to slope gradient in this study, this confirmed the previous findings by Zhang *et al*.^34^ and Zhang *et al*.^35^ while inconsistent with the results of Zhang *et al*.^21^ and Xiao *et al*.^11^. The experiment conditions varied among different research may resulted in such differences. The natural, undisturbed soil samples and the disturbed soil samples were used in Zhang *et al*.^21^ and in our study, respectively. The soil detachment capacity of disturbed soil samples was notably higher (1 to 23 times) than those of the natural, undisturbed soil samples^21,34^, suggesting the disturbed soil samples were easier eroded by overland flow. Xiao *et al*.^11^ tried to simulate the true detachment process of concentrated flow on slope during rill development, while this study mainly focuses on the effect of concentrated flow on the soil surface. Therefore, many rill subprocesses (e.g. headcut erosion, sidewall failure sidewall failure)^36^ would act as the sediment source for detachment, which of course influenced the relationship slope gradient and flow rate and the soil detachment.

All the flow hydrodynamic parameters were significant positive correlations with the soil detachment capacity in this study. The relationship between soil detachment capacity and flow hydrodynamic parameters could be well describe by both linear function and power function, of which the power function relationship performed better in generally. These results were in line with previous studies undertaken on the different types of soil form different area^14,21^. The values of flow hydrodynamic parameters under low flow rate and gentle slope gradient were almost equal to or even less than the critical hydrodynamic parameter values (Table 4), for which the soil detachment capacity should be near to zero or negative according to the linear function relationship. However, the soil still eroded by the overland flow under these conditions, which could be weaken the performance the linear function relationship. The ‘roll wave’ occurred under low flow rate and gentle slope gradient conditions and the peak of wave generated lots of soil loss according to our observation during experiment. Our study confirmed stream power as a good indicator of flow erosivity and it was also identified as the best hydrodynamic parameter for detachment prediction, which was in accordance with previous findings of other researchers^11,19^.

**Table 4:** The flow properties used in this research.

Slope gradient (%)	Flow rate (L min^−1^)	Shear stress (Pa)	stream power (N m^−1^ s^−1^)	Unit stream power (m s^−1^)	E (m)
5	5	0.967	0.180	0.016	0.003
	10	1.535	0.372	0.021	0.005
	15	1.774	0.545	0.027	0.007
	20	2.084	0.696	0.029	0.008
	25	2.433	0.891	0.032	0.010
	30	2.773	1.066	0.033	0.011
10	5	1.491	0.376	0.044	0.004
	10	1.959	0.688	0.061	0.007
	15	2.769	1.067	0.067	0.009
	20	3.474	1.425	0.071	0.011
	25	3.792	1.773	0.081	0.013
	30	4.147	2.124	0.089	0.016
15	5	1.561	0.491	0.081	0.006
	10	2.564	1.110	0.112	0.011
	15	3.596	1.612	0.116	0.012
	20	3.867	2.130	0.143	0.017
	25	4.373	2.632	0.156	0.020
	30	4.636	3.208	0.179	0.026
20	5	2.091	0.691	0.113	0.006
	10	3.338	1.334	0.137	0.009
	15	4.347	2.004	0.158	0.012
	20	5.284	2.695	0.174	0.015
	25	6.254	3.526	0.193	0.018
	30	6.797	4.204	0.212	0.022
25	5	2.070	0.932	0.190	0.011
	10	3.789	1.777	0.198	0.012
	15	5.097	2.658	0.220	0.015
	20	5.863	3.425	0.247	0.019
	25	6.896	4.219	0.259	0.021
	30	7.422	5.281	0.301	0.028
30	5	2.638	0.939	0.178	0.007
	10	3.948	1.950	0.247	0.013
	15	5.139	2.981	0.290	0.018
	20	6.820	4.185	0.307	0.021
	25	7.896	5.023	0.318	0.022
	30	8.035	6.176	0.384	0.032

In this study, the erodibility value based on shear stress was 0.780 s m^−1^, and the critical shear stress was 1.459 Pa, both of them were within the range of the values reported by Geng *et al*.^37^, who researched 36 types of soil in the eastern China. The mean erodibility value and the mean critical shear stress was 0.511, 0.226, 0.174, 0.157, 0.127 and 0.033 s m^−1^ and 2.73 Pa, 2.92, 3.01, 2.67, 2.51 and 3.81 Pa for northwest Loess Plateau, south mountains and hills, north mountains and hills, Sichuan Basin and surrounding mountains and hills, northeast low mountains and hills and Yunnan-Guizhou Plateau, respectively^37^, suggesting the erodibility value in this study was larger than and the critical shear stress was less than all of them. This could attribute to the characteristic of a low degree of development, parent materials of the soil in this study^33^, while well development for the soil test in Geng *et al*.^37^. The erodibility value in this study was even larger than and the critical shear stress was less than those for the northwest Loess Plateau, respectively, where is susceptible to severe water erosion and has been ranked as being most severely eroded area^38^. Thus, there has a huge potential for the soil in the Tibet Plateau eroded by the water erosion when enough runoff exiting. More overland flow will occur under the condition of climate warming^2,7^. In addition, the climate warming negatively impacted soil aggregate stability, resulting in a significant decrease in MWD and GMD^3^. The soil aggregation plays an important role in soil erodibility, many researchers have reached the consensus that the indicator of structural stability of soil aggregates is in close relation to soil erosion^39–41^. The more stable the soil aggregate is, the higher resistance for the soil to water erosion^39,42^. Thus, climate warming could aggravate soil erosion by both increasing runoff and weakening the soil structure. More attention should be payed to the water erosion process and mechanism in the Tibet Plateau area in the future.

Our results are only based on one soil type, whereas there have diversity soil types existed in the Tibet Plateau area^33^. The soil type with various texture characteristics and physicochemical property have huge influence on the soil detachment capacity as mentioned above^37^. This study focused on the effect of overland flow on the soil surface to understand the soil detachment capacity, rill erodibility and critical hydrodynamic parameter, but not study the real rill erosion process for the soil in the Tibet Plateau area^43,44^. Thus, The real rill erosion of other types of soil^45^, the influence of freeze–thaw effects and the snowmelt erosion process should be investigated in the future for better understanding the overland erosion characteristic in the Tibet Plateau area.

## Materials and Methods

### Soils

The uppermost 15-cm layer soil was collected from Mangkang County (28°37′–30°20′N and 98°00′–99°05′E), which is located in the southeast of Tibet Autonomous Region, China. The annual average precipitation and temperature is 350–450 mm and 10 °C, respectively. The soil is classified as Cambosol or Inceptisol according to the Chinese Soil Taxonomy or USDA Soil Taxonomy, respectively. The soil comprise 67.74% sand, 18.45% silt, 13.82% clay, with a pH value of 7.03 and 13.06 g kg^−1^ of organic matter content. The collected soil was air-dried and gently screened through a 5-mm sieve for removing the impurities such as roots and gravel in the soil^46^. The soil was packed in three layers with 1, 2 and 2 cm per layer from top to bottom, the soil amount of each layer was calculated before packing, and each packed soil layer was coarsened lightly before the next layer was packed to achieve the desired uniform target bulk density (1.40 g cm^−3^ according to the field data) in the steel cylinder (105 mm-diameter and 50 mm-depth).The soil sample was watered to saturation for experiment.

### Experimental design

**Figure 3:**
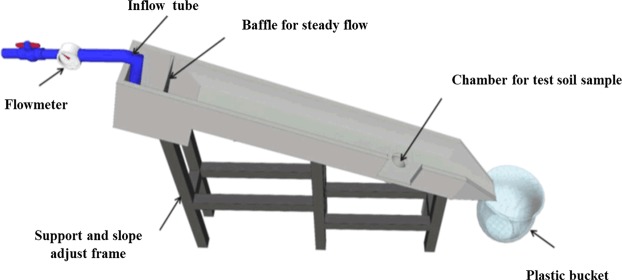
Schematic representation of the hydraulic flume.

An series of laboratory experiments were performed by using a slope-adjustable steel hydraulic flume (4 m length, 0.2 m depth, 0.4 m width) (Fig. 3), which was similar to that described by Zhang *et al*.^21^ and Wang *et al*.^14^. The test soil was glued on the surface of the flume bed to simulate the natural soil surface condition and maintain the same roughness of the flume bed during the experiment^21^. Thirty-six combinations of six flow rates (5, 10, 15, 20, 25 and 30 L min^−1^) and six slope gradients (8.74%, 17.63%, 26.79%, 36.40%, 46.63 and 57.73%) were investigated, these slope gradients are chosen according our field investigation and different flow rates are used for obtaining different flow conditions. The flow shear stress, stream power, unit stream power and unit energy of water-carrying section ranged from 0.967 to 8.035 Pa, from 0.180 to 6.176 N m^−1^ s^−1^, from 0.016 to 0.384 m s^−1^and from 0.289 to 3.178 cm, respectively (Table 3).

Before each test, the flume was set at the designed slope gradient and flow supplied by a pipe had been adjusted to obtain the designed flow rate (the error was less than 5%). Flow velocity between the cross sections at the transects 0 to 1 m away from the soil chamber was also determined before test^46^. Flow velocity was obtained by using the dye-tracing method, the mean flow velocity could be corrected after multiplying a correction factor (0.67 in this research) because the travel time of the dye tracer was the surface velocity of the flow which was always larger than the mean flow velocity^47^. Five replicates of flow velocity were measured for each test. The pipe was moved out of the flume after measuring the flow velocity, and the saturated soil sample was inserted into the soil chamber to make sure that the surface of the sample was in line with the flume bed. The soil sample was covered by a panel to prevent soil sample be detached until the overland flow be in a steady state, and the test was initiated. The test was stopped when the scouring depth reached about 2 cm to avoid the boundary effects from steel cylinder^14^. All the runoff and sediment were collected by using the 30-L plastic containers over the test period. Sediment amount for each test were determined by oven-drying the samples at 105 °C more than 24 h in the laboratory.

The particle size distribution was analyzed by using a TopSizer laser diffraction device (Zhuhai OMEC instrument Co., Ltd., China). The pH value was determined by using the Rex Electric Chemical PHS-3E precision acidity meter (Shanghai Precision Scientific Instrument Co., Ltd, China). The soil organic matter was tested by using potassium dichromate oxidation-external heating method^48^.

### Equations and data analysis

The soil detachment rate is obtained under clear overland flow without any sediment load condition, so it can define as soil detachment capacity, *D*_*r*_, (kg m^−2^ s^−1^), which can be can be calculated using Equation 2:

2 $${D}_{r}=\frac{E}{St}$$

where *E* is the sediment amount for each test (kg); *S* is the cross-section area of the soil sample (m^2^) and *t* is the test duration (s).

The Equations 3, 4, 5 and 6 are used for calculating shear stress, stream power, unit stream power and unit energy of water-carrying, respectively.

3 $$\tau =\rho gRJ$$

4 $$\omega =\tau V=\rho gqJ$$

5 $$P=VJ$$

6 $$E=\frac{a{V}^{2}}{2g}+h$$

where *τ* is shear stress (Pa or N m^−2^); *ρ* is the density of water (kg m^−3^); *g* is the gravitational acceleration (m s^−2^); and *R* is the hydraulic radius, which was considered equal to the flow depth (*h*) under the overland flow condition (m); *J* (m m^−1^) is the slope gradient; *ω* is stream power (N m^−1^ s^−1^); *q* is the unit discharge per unit width (m^2^ s^−1^); *P* is the unit stream power (m s^−1^); *V* is flow velocity (m s^−1^); *E* is the unit energy of water-carrying section (m) and *a* is the correction factor for kinetic energy, usual equal to 1^49^.

The flow depth (*h*, m) is only in several millimeter and often in dynamic condition, so it very difficult to monitor accurately. Therefore, the mean flow depth is calculated by assuming a uniform concentrated flow along the flow width, which can be can be calculated using Equation 7:

7 $$h=\frac{O}{VW}$$

where *O* is the flow rate (m^3^ s^−1^); w is the flow width (m); and *V* is the mean calculated flow velocity (m s^−1^).

### Statistical analysis

The relationships of soil detachment capacity with flow rate, slope gradient, shear stress, stream power, unit stream power and unit energy of water-carrying section were established via regression analysis by Excel 2016. The correlation between soil detachment capacity with runoff rate and slope gradient was analyzed via Pearson correlation coefficient by SPSS 21.0 (SPSS Inc., Chicago, IL, USA). The values of the statistical parameters of *R*^2^, *NRMSE* and *NSE* were used as indicators of evaluate the efficiency of equations in this study. The value of *R*^2^, *NRMSE* and *NSE* can be calculated using Equations 8, 9 and 10.

8 $${R}^{2}=\frac{[{\sum }_{i=1}^{n}({X}_{o,i}-{X}_{o,avg})({X}_{p,i}-{X}_{p,avg})]}{{\sum }_{i=1}^{n}{({X}_{o,i}-{X}_{o,avg})}^{2}{\sum }_{i=1}^{n}{({X}_{p,i}-{X}_{p,avg})}^{2}}$$

9 $$NRMSE=\frac{\sqrt{\frac{{\sum }_{i=1}^{n}{({X}_{o,i}-{X}_{p,i})}^{2}}{n}}}{{X}_{o,avg}}$$

10 $$NSE=1-\frac{{\sum }_{i=1}^{n}{({X}_{o,i}-{X}_{p,i})}^{2}}{{\sum }_{i=1}^{n}{({X}_{o,i}-{X}_{o,avg})}^{2}}$$

where *X*_*o*,*i*_ is the observation value for *i*; *X*_*p*, *i*_ is the calculation value for *i*; *X*_*o*,*avg*_ is the mean observation value; *X*_*p*,*avg*_ is the mean calculation value. NSE reflects the relative magnitude of the residual variance compared with the variance of the observation value. Values > 0.7, 0.4 < NSE ≤0.7 and <0.4 are generally viewed as good performance, satisfactory performance and unacceptable performance, respectively^50^.

## References

[1] Hansen J (2006). Global temperature change. P Natl Acad Sci.

[2] Freppaz M, Celi L, Marchelli M, Zanini E (2008). Snow removal and its influence on temperature and N dynamics in alpine soils (Vallée d’Aoste, Northwest Italy). Journal of Plant Nutrition and Soil Science..

[3] Guan S (2018). Climate warming impacts on soil organic carbon fractions and aggregate stability in a Tibetan alpine meadow. Soil Biology and Biochemistry..

[4] IPCC. Climate Change 2013: The Physical Science Basis. *Contribution of Working Group I to the Fifth Assessment Report of the Intergovernmental Panel on Climate Change*. (Cambridge University Press, 2013).

[5] Ren G (2012). Recent progress in studies of climate change in China. Advances in Atmospheric Sciences.

[6] Hu YG (2017). Climate change affects soil labile organic carbon fractions in a Tibetan alpine meadow. J Soils Sediments..

[7] Ban YY, Lei TW, Chen C, Liu ZQ (2016). Study on the facilities and procedures for meltwater erosion of thawed soil. International Soil and Water Conservation Research..

[8] Ban YY, Lei TW, Liu ZQ, Chen C (2017). Comparative study of erosion processes of thawed and non-frozen soil by concentrated meltwater flow. Catena..

[9] Ellison WD (1947). Soil erosion studies: Part I. Agricultural Engineering..

[10] Zhang GH, Liu GB, Tang KM, Zhang XC (2008). Flow detachment of soils under different land uses in the Loess Plateau of China. Transactions of the American Society of Agricultural and Biological Engineers..

[11] Xiao H (2017). Response of soil detachment rate to the hydraulic parameters of concentrated flow on steep loessial slopes in on the Loess Plateau of China. Hydrological Processes..

[12] Capra A, Porto P, Scicolone B (2009). Relationships between rainfall characteristics and ephemeral gully erosion in a cultivated catchment in sicily (italy). Soil & Tillage Research..

[13] Gong JG (2011). An experimental study on dynamic processes of ephemeral gully erosion in loess landscapes. Geomorphology..

[14] Wang Y (2017). Modelling soil detachment of different management practices in the red soil region of China. Land Degradation & Development..

[15] Nearing MA, Foster GR, Lane LJ (1989). A process-based soil erosion model for USDA-water erosion prediction project technology. Transactions of the American Society of Agricultural Engineers..

[16] Zhang GH, Liu YM, Han YF, Zhang XC (2009). Sediment Transport and Soil Detachment on Steep Slopes: II. Sediment Feedback Relationship. Soil Sci. Soc. Am. J..

[17] Wang B, Zhang GH, Yang YF, Li PP, Liu JX (2018). The effects of varied soil properties induced by natural grassland succession on the process of soil detachment. Catena..

[18] Zhang KL, Zhang ZM (2000). Erosion and sediment delivery in rills on steep loess slope. Progress in Natural Science..

[19] Wang DD, Wang ZL, Shen N, Chen H (2016). Modeling soil detachment capacity by rill flow using hydraulic parameters. Journal of Hydrology..

[20] Jiang FS (2018). Rill erosion processes on a steep colluvial deposit slope under heavy rainfall in flume experiments with artificial rain. Catena..

[21] Zhang GH, Liu BY, Liu GB, He XW, Nearing MA (2003). Detachment of undisturbed soil by shallow flow. Soil Science Society of America Journal..

[22] Morgan RPC (1998). The European Soil Erosion Model (EUROSEM): A dynamic approach for predicting sediment transport from fields and small catchment. Earth Surface Processes and Landforms..

[23] Misra RK, Rose CW (1996). Application and sensitivity analysis of process-based erosion model GUEST. European Journal of Soil Science..

[24] Wang B, Zhang GH, Shi YY, Zhang XC (2014). Soil detachment by overland flow under different vegetation restoration models in the Loess Plateau of China. Catena.

[25] Hanson GJ, Robinson KM (1993). The influence of soil moisture and compaction on spillway erosion. Transactions of the ASAE.

[26] Sheridan GJ, So HB, Loch RJ, Walker CM (2000). Estimation of erosion model erodibility parameters from media properties. Australian Journal of Soil Research.

[27] Geng R, Zhang GH, Ma QH, Wang H (2017). Effects of landscape positions on soil resistance to rill erosion in a small catchment on the Loess Plateau. Biosystems Engineering.

[28] Liu QJ, Wells RR, Dabney SM, He JJ (2017). Effect of Water Potential and Void Ratio on Erodibility for Agricultural Soils. Soil Science Society of America Journal.

[29] Wang JG (2012). Predicting physical equations of soil detachment by simulated concentrated flow in Ultisols (subtropical China). Earth Surf. Proc. Land..

[30] Xiao H (2017). Developing equations to explore relationships between aggregate stability and erodibility in Ultisols of subtropical China. Catena.

[31] Guo MM, Wang WL, Kang HL, Yang B (2018). Changes in soil properties and erodibility of gully heads induced by vegetation restoration on the Loess Plateau, China. Journal of Arid Land.

[32] Zhu Guangyu, Tang Zhuangsheng, Shangguan Zhouping, Peng Changhui, Deng Lei (2019). Factors Affecting the Spatial and Temporal Variations in Soil Erodibility of China. Journal of Geophysical Research: Earth Surface.

[33] Fang HB (2015). Soil taxonomy and distribution characteristics of the permafrost region in the Qinghai-Tibet Plateau, *China*. J. Mt. Sci..

[34] Zhang GH, Liu BY, Nearing MA, Huang CH, Zhang KL (2002). Soil detachment by shallow flow. Transactions of the American Society of Agricultural Engineers..

[35] Zhang QW, Lei TW, Zhao J (2008). Estimation of the detachment rate in eroding rills in flume experiments using an REE tracing method. Geoderma..

[36] Zhang XC, Li ZB, Ding WF (2005). Validation of WEPP sediment feedback relationships using spatially distributed rill erosion data. Soil Science Society of America Journal.

[37] Geng R, Zhang GH, Ma QH, Wang LJ (2017). Soil resistance to runoff on steep croplands in Eastern China. Catena..

[38] Shi H, Shao MA (2000). Soil and water loss from the Loess Plateau in China. Journal of Arid Environments..

[39] Barthès B, Roose E (2002). Aggregate stability as an indicator of soil susceptibility to runoff and erosion: validation at several levels. Caten..

[40] Dimoyiannis D, Valmis S, Danalatos NG (2006). Interrill erosion on cultivated Greek soils: modeling sediment delivery. Earth Surf. Proc. Land..

[41] Shi ZH, Yan FL, LI L, Li ZX, Cai CF (2010). Interrill erosion from disturbed and undisturbed samples in relation to topsoil aggregate stability in red soils from subtropical China. Catena..

[42] Ding WF, Zhang XC (2016). An evaluation on using soil aggregate stability as the indicator of interrill erodibility. J. Mt. Sci-Engl..

[43] Sun BY (2018). An analysis of soil detachment capacity under freeze-thaw conditions using the Taguchi method. Catena.

[44] Gao XF, Li FH, Chen C, Ban YY, Gao Y (2019). Effects of thawed depth on the sediment transport capacity by melt water on partially thawed black soil slope. Land Degrad. Dev..

[45] Xiao H (2018). Quantifying contributions of slaking and mechanical breakdown of soil aggregates to splash erosion for different soils from the loess plateau of china. Soil and Tillage Research..

[46] Zhang GH, Luo RT, Cao Y, Shen RC, Zhang XC (2010). Correction factor to dye-measured flow velocity under varying water and sediment discharges. J. Hydrol..

[47] Li G, Abrahams AD, Atkinson JF (1996). Correction factors in the determination of mean velocity of overland flow. Earth Surf. Proc. Land..

[48] Liu, G. S. Soil physical and chemical analysis and description of soil profiles. (Standards Press of China, 1996).

[49] Kang HL (2016). Effect of gravel on runoff and erosion characteristics on engineering accumulation slope in windy and sandy area, northern China. Transactions of the Chinese Society of Agricultural Engineering..

[50] Wu B, Wang ZL, Shen N, Wang S (2016). Modelling sediment transport capacity of rill flow for loess sediments on steep slopes. Catena..

